# Borderline pattern - an indicator of the severity of personality disorders

**DOI:** 10.1192/j.eurpsy.2025.1907

**Published:** 2025-08-26

**Authors:** A. Bogdanovska Toskic, S. Arsova Hadzi-Angjelkovska

**Affiliations:** 1 Psychiatry, General Hospital, Kumanovo; 2 University Clinic of Psychiatry, Skopje, North Macedonia

## Abstract

**Introduction:**

The borderline pattern is an optional diagnostic specification in ICD 11 that includes the diagnostic criteria for borderline disorder. With the introduction of the dimensional diagnosis of personality disorders by grading the severity of the disorder and determining the pathological traits, a debate has been opened about the need for the borderline pattern specifier.

**Objectives:**

The objective of the study is to determine the relationship of the borderline pattern with the level of personality functionality and its role in the differentiation of patients with and without personality disorder.

**Methods:**

An analytical cross-sectional study was conducted to determine the degree of severity of personality disorder and borderline pattern in patients diagnosed with personality disorder according to ICD 10 compared to healthy individuals. LPFS-BF-2.0 was used to assess the degree of personality functioning, and BPS was used to assess the existence and intensity of a borderline pattern.

**Results:**

Two groups between the ages of 18 and 65 were included in the study, the first of 17 people (*N* 17, 41% women) (mean age 34.29, SD 13.85) with a diagnosed personality disorder according to ICD 10 and the second a control group of 22 healthy subjects (*N* 22, 59% female) (mean age 33.18 years, SD 11.76). The first respondent group had LPFS-BF-2.0 scores of 29.88 (SD 4.93) and BPS 38.06 (SD 7.3). The control group had LPFS-BF-2.0 scores of 17.22 (SD 3.99) and BPS 20.09 (SD 6.05). A high correlation was found between LPFS-BF-2.0 and BPS, *r(39)=0.82 p=0.0000.* The differences between the two groups were high both according to the level of personality functionality *t(37)=8.849 p<0.00001* and according to the intensity of the borderline pattern *t(37)=8.401 p<0.00001.*

**Conclusions:**

According to the obtained results for the high correlation between the intensity of the borderline pattern and the level of functionality of the person, it is concluded that the borderline pattern is an indicator of the degree of severity of a personality disorder. The significant difference between the levels of personality functionality and the borderline pattern in both groups can be used in the differential diagnosis of persons with and without personality disorder and other mental disorders, which would have implications for the therapy plan.

**Disclosure of Interest:**

None Declared

